# The effects of perceived sport environment on sport gains of Chinese university students: chain mediation between physical activity behavior and sport learning self-efficacy

**DOI:** 10.3389/fpsyg.2024.1466457

**Published:** 2024-12-10

**Authors:** Liang Wu, Jia Gao, Jun Xiang

**Affiliations:** School of Physical Education and Health, Zhaoqing University, Zhaoqing, China

**Keywords:** perceived physical education environment, physical education gains, physical activity behavior, sport learning self-efficacy, mediating role, college students

## Abstract

**Introduction:**

Sports gains reflect the sports development of college students, which is a direct reflection of the quality of school sports work, while the perception of sports environment, physical activity behavior, and self-efficacy in sports learning are closely related to their sports gains, which directly affects their effectiveness and interest in sports learning.

**Objective:**

To investigate the effects of perceived sports environment on Chinese college students’ sport gains, and to verify the mediating roles of physical activity behavior and self-efficacy in sport learning.

**Methods:**

A survey of 2,207 Chinese university students was conducted using the sport environment perception scale, sport gain scale, physical activity behavior scale, and sport learning self-efficacy scale.

**Results:**

(1) There were significant differences in age and gender between college students’ perceptions of sport environment, sport gains, sport exercise behavior, and sport learning self-efficacy, and the mean scores of each scale were better for male students than for female students. (2) Perception of sports environment was significantly positively correlated with sports gain, and perception of sports environment had a direct positive effect on sports gain. In addition, sport environment perception positively predicted physical activity behavior and sport learning self-efficacy; physical activity behavior significantly predicted sport learning self-efficacy and sport gains; physical learning self-efficacy was a significant positive predictor of sport gains. (3) Physical activity behavior and physical learning self-efficacy played a significant chained mediating role between perceived physical environment and physical gains. These results are important for promoting physical education learning outcomes among college students and provide a theoretical basis for developing interventions. However, there are limitations to this study, such as the specificity of the sample and the self-reported data used. Future research could expand the sample and utilize multiple assessment methods to validate these results.

## 1 Introduction

Sports gains serve as the indicators of the athletic development status of college students, encompassing improvements in intelligence, physical ability, interpersonal ability, and growth goals. These gains manifest in three primary domains: cognitive advancements, practical skills acquisition, and social adaptability (Gong, 2021). General Secretary Xi Jinping emphasized during the National Education Conference that fostering college students’ experiences and perceptions of gains related to physical fitness improvement, skill acquisition, and character development constitutes a vital objective of their sports participation. This, in turn, serves as a key metric for evaluating the quality of college sports programs (Wang and Li, 2024; Zhou et al., 2024). The research indicates that college sports represent the culmination of the school sports experience for most students, acting as a crucial link between school-based and community sports. Participation in sports not only enhances sports gains but also cultivates beneficial physical exercise habits, thereby ensuring the holistic well-being of college students (Cronin et al., 2018). The process of developing college students’ physical ability and mastering skills is also the process of promoting college students’ sound personality and integrating into the social group (Wang and Li, 2024). Therefore, it is important to clarify the sports gains of college students’ sports participation in the development of physical ability, mastering of skills, and spiritual shaping.

Research has also shown that school environment is reflected in elements such as curriculum design, teacher guidance, support services, and cultural incentives (Gong et al., 2021). Perceptions of the environment are closely tied to students’ awareness of the resources available to them, playing a critical role in personal development and learning outcomes (Dewaele and Li, 2021). The overall growth of students hinges on their ability to assimilate into the school environment and actively engage in social interactions. This is particularly salient in the context of college athletics, where research indicates that students’ perceptions of the athletic environment significantly influence their participation levels and resultant gains (Nthangeni et al., 2021). The autonomy and adaptability inherent in college athletic programs, coupled with faculty mentorship, facility access, and institutional policies, highlight the importance of the organizational environment in shaping student development (Simón-Chico et al., 2023). A genuine awareness of environmental conditions is indispensable to optimize the impact of the environment on sport gains, physical activity behaviors, and sport learning self-efficacy, which highlights the synergistic effects between the university sport environment, students’ sport participation, and sport gains (Castillo et al., 2020). Therefore, the present study aimed to investigate the effects of perceived sport environment on sport gains of Chinese college students and to investigate the mediating role of physical activity behavior and sport learning self-efficacy in this relationship. The autonomy and adaptability inherent in college athletic programs, coupled with faculty mentorship, facility access, and institutional policies, highlight the importance of the organizational environment in shaping student development.

## 2 Literature review and research hypotheses

### 2.1 Sports environment perception and sports gaining

Research has pointed out that there is a significant positive relation between college students’ sports environment perception and college students’ sports gains (Gong et al., 2021). Scholars also point out that the ability to provide students with a greater sense of gain is the basic yardstick for evaluating the effectiveness of school physical education development (Fu, 2012). The extent to which students engage in meaningful educational activities throughout their school years is recognized as a critical factor influencing their overall development. Moreover, a supportive school environment significantly affects the quality of student participation, thereby shaping their individual growth, learning efficacy, and overall gains (Guo et al., 2022). The school sports ecological environment, which comprises physical education teachers, students, school leaders, campus sports culture, sports systems, and facilities, constitutes a micro-system that is intimately connected to students’ sports practices. This environment plays a pivotal role in fostering student participation in sports, reinforcing healthy athletic behaviors, and enhancing sports-related gains (Gong, 2021). Consequently, it can be inferred that a correlation exists between college students’ perceptions of their sports environment and their sports gains. Therefore, we proposed hypothesis 1: College students’ perceptions of their sports environment have a positive predictive effect on their sports gains.

### 2.2 Mediating role of physical activity behavior

One of the mediating mechanisms in this study is the mediating role of physical activity behavior. Physical activity behavior plays an important mediating role between the perception of sports environment and sports gains. Physical activity behavior is a form of activity that can improve one’s physical fitness and make one feel enjoyable. Regular participation in physical activity not only contributes to the enhancement of physical fitness but also fosters skill development, boosts self-esteem, and alleviates stress (Liang, 1994). The Chinese university environment is characterized by academic stress and a sedentary lifestyle, making the role of physical activity particularly important (Yang et al., 2013). Research has shown that the perception of college students’ sports environment is significantly positively correlated with college students’ participation in physical activity (Gong et al., 2021). Furthermore, the college sports environment significantly influences college students’ participation in physical activity through its inherent attributes of temporality, values, and mechanisms. This environment facilitates students’ engagement in physical activities and meets their developmental needs through effective policy guidance and cultural reinforcement (Vats, 2023). In the case that the internal psychological mechanism of individuals is difficult to grasp., the synergy of multiple constraints is difficult, and the improvement of the external environment is feasible, we should consider the combination of the environmental situation, process situation, and gain situation of physical activity participation, and comprehensively study the system of physical activity participation, the process of physical activity participation, and the results of physical activity participation (Gong, 2021). It can be seen that the scholars’ research emphasizes how positive perceptions of the physical education environment influence the necessity of students’ actual participation in physical activity.

Research has also shown that there is a positive link between physical activity behaviors and sport gains (Yıldız et al., 2021). First, engaging in physical activity has been shown to improve mental acuity, memory retention, and overall cognitive functioning. For instance, aerobic exercises such as running or biking promote neuroplasticity and neurogenesis, which positively influence cognitive performance. This, in turn, contributes to improved academic performance and the development of cognitive skills in physical education (Ferrer-Uris et al., 2022). Second, the relationship also extends to emotional well-being, as physical activity triggers the release of endorphins, reduces stress and anxiety levels, and promotes a positive mental state (Mashhadi et al., 2021). Third, the literature also emphasizes the key role of physical activity in improving social adjustment as participation in team sports or group exercise promotes social interaction, communication skills and teamwork (Panda, 2024). As can be seen, all three of the above points show results that emphasize the intricate interactions between physical activity behaviors and different sport gains, covering cognitive, affective, and social dimensions, and emphasizing the overall benefits of an active lifestyle from sport. Therefore, this study hypothesizes that physical activity behaviors mediate the relationship between perceived sport environment and sport gains, therefore, we proposed Hypothesis 2: physical activity behaviors mediate between perceived sport environment and sport gains.

### 2.3 Mediating role of sport learning self-efficacy

The second mediating mechanism of this study is the mediating role of sport learning self-efficacy. Sport learning self-efficacy plays an important mediating role between sport environment perception and sport gains. Scholars have pointed out that there is a positive association between perceived college sport environment and sport learning self-efficacy (Wang et al., 2020). Students who perceive a positive and supportive sport environment are more likely to develop higher levels of self-efficacy in their sport learning and performance abilities, and such positive perceptions of the environment cultivate a sense of competence and self-confidence, which influences students’ perception of their own beliefs about successfully participating and excelling in physical activity (Han et al., 2022). In addition, Bandura’s self-efficacy theory suggests that an individual’s perception of the environment can have a significant impact on his or her sense of self-efficacy. A positive, inclusive sports environment that includes supportive teachers, appropriate facilities, and engaging activities fosters a sense of ownership and control, which in turn enhances students’ self-efficacy and influences their motivation and effort in sports learning, which in turn facilitates their sports acquisition (Kok et al., 2020). In addition, intrinsic factor (such as identity and motivation) could contribute in making positive sports environment (Lupo et al., 2017a; Lupo et al., 2017b). It is evident that the positive relationship between perceived sport environment and sport learning self-efficacy lays the foundation for subsequent sport gains by fostering a mindset conducive to skill development, perseverance, and overall achievement. Therefore, this study hypothesized that Sport learning self-efficacy plays a mediating role between perceived college physical education environment and sport gains. Therefore, we proposed Hypothesis 3: Sport learning self-efficacy has a mediating role between university physical education environment perception and physical education gain.

### 2.4 Chain mediation between physical activity behavior and sport learning self-efficacy

Physical activity behavior and sport learning self-efficacy may have a chain mediating role between perceived college sport environment and sport gains. Scholars have observed that individuals who engage regularly in physical activity develop higher levels of self-efficacy in sport learning. Scholars have noted that individuals who regularly participate in physical activity develop higher levels of self-efficacy in sport learning, and that active participation in physical activity provides individuals with tangible experiences and accomplishments that contribute to their self-efficacy and their belief in their ability to master and improve their sport skills (Ten Brummelhuis et al., 2022). This period is associable to previous studies on collegiate/university student-athletes for which younger and elite athletes demonstrated to have high identification in their (student-athletes) role and motivation for better succeeded in dual career (Lupo et al., 2017b). Moreover, a supportive university sport environment, including well-equipped facilities, qualified instructors, and a variety of engaging activities, can inspire and motivate individuals to actively participate in sport, which can positively impact their sport learning self-efficacy (Kok et al., 2020; Gong, 2021). The positive influence between the perceived college sport environment, physical activity behavior, sport learning self-efficacy, and subsequent sport gains highlights the integral role that the college sport environment plays in shaping an individual’s well-rounded and rewarding sport experience (Hauser et al., 2022). It is evident that the relationship between physical activity behavior and sport learning self-efficacy plays a pivotal role in moderating the impact of the college sport environment on overall sport gains. Therefore, this study hypothesizes that physical activity behavior and sport learning self-efficacy constitute a chain mediation between perception of the college sport environment and sport gains, and that perception of the college sport environment enhances sport gains by increasing individuals’ physical activity behavior and sport learning self-efficacy. Therefore, we proposed Hypothesis 4: physical activity behavior and sport learning self-efficacy have a chain mediating role between university sport environment perception and sport gain.

In summary, scholars have pointed out the positive association between sport environment perception and sport gain, and emphasized the intermediary and chain intermediary roles of physical activity behavior and sport learning self-efficacy in this relationship, and the above findings provide the theoretical basis for the hypotheses of this study. In addition, previous studies found that boys and girls show differences in the levels of sport-related variables (e.g., Alcaraz-Muñoz et al., 2023; O’Connor et al., 2023; Lupo et al., 2017a). Boys tend to engage in higher levels of physical activity and organized sports, often influenced by societal expectations that encourage competitiveness and physicality. In contrast, girls may participate less frequently, partly due to perceptions of the sport environment that can be less welcoming or supportive, leading to feelings of exclusion (Alcaraz-Muñoz et al., 2023). Additionally, girls often report lower sport learning self-efficacy, which can stem from a lack of encouragement or role models in sports. While both genders can experience benefits from physical activity, such as improved health and social connections, boys often report greater confidence in their abilities and a stronger belief in their potential to succeed in sports (Carr and Volberding, 2014). In addition, Studies have reported that different cognitive, emotional, and physical development are influential for different age groups participating in sports (Evmenenko et al., 2022). Therefore, it is necessary to examine the gender and age differences in physical activity behavior, perceptions of the sport environment, sport-related gains, and sport learning self-efficacy, and to consider gender as control variable in the research.

**Figure 1:**
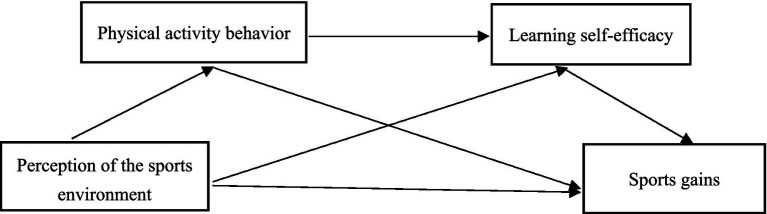
Research framework diagram.

Therefore, the research architecture concepts of this study were established (Figure 1): (1) to test the effect of sport environment perception on college students’ sport gains; (2) to examine the mediating role of physical activity behavior between sport environment perception and college students’ sport gains; (3) to examine the mediating role of sport learning self-efficacy between sport environment perception and college students’ sport gains; and (4) to examine the mediating role of physical activity behavior and sport learning self-efficacy in the chain mediating role between perceived sport environment and college students’ sport gains. Physical activity behavior Learning self-efficacy.

## 3 Objects and methods of research

### 3.1 Collection procedures

Three days prior to the formal signing of the informed consent form, members of the research team asked staff (physical education teachers) to send the informed consent form to each class group (51 classes, totaling 2,500 students) via WeChat. This allowed college students to familiarize themselves with information related to the study’s purpose, content, confidentiality agreement, and contact details of the researchers. They were also informed that they would need to formally fill out the informed consent form and the survey questionnaire during the official physical education class.

On the day of the formal testing, before signing the informed consent form, the research team staff and physical education teachers reiterated the content and requirements of the informed consent form to ensure that students understood the testing content and the questionnaire format. They also addressed any questions raised by the participants. Subsequently, the informed consent form was signed on-site with the voluntary participation of the college students.

Finally, staff conduct face-to-face survey filling during physical education classes. Due to factors such as the large number of students selected from classes and varying physical education class times, the study ultimately collected basic information about the participants (such as gender, grade, etc.), along with evaluations of their perceptions of the physical education environment, sports benefits, sports participation behavior, and self-efficacy in sports learning. This research survey took a total of two months to complete.

### 3.2 Participants

Inclusion criteria for subjects before questionnaire distribution: (1) physically and mentally healthy people; (2) college students; (3) no major diseases, subjects who will not meet the above criteria will be excluded from the study. After the questionnaires were recovered, they were excluded according to the following criteria: (1) missing data; (2) inconsistent responses; and (3) those with limited physical activity. After collation 2,207 valid questionnaires were recovered for this study (293 subjects were excluded due to missing data on the main variables and inclusion criteria), with a recovery rate of 88.28%. The sample size was computed *a priori* by G∗Power 3.1 (Faul et al., 2007), using the power of (1-*β*) = 0.95, medium effect size |𝜌| = 0.3 of the expected variable-correlations and a two-tail test. The recommended sample size by G∗Power calculation was 134 and we obtain reliable statistical calculations. The age range of the participants was 17–23 years (*M* age = 19.27, SD age = 1.18), of which 903 (37.5%) were male and 1,304 (54.2%) were female. There were 902 (37.5%) freshmen, 985 (40.9%) sophomores, 260 (10.8%) juniors, and 60 (2.5%) seniors, and there were no significant differences between the variables for different genders and grades. In addition, this study was supported and approved by the Institutional Review Board of Zhaoqing College. All participating Chinese college students signed an informed consent form. Among other things, the informed consent form described the purpose and process of the study, among other things, and also included information such as a confidentiality agreement, the principle of voluntary participation, and the contact information of the researcher, as well as controlling for variables such as gender and grade level of the subjects.

### 3.3 Research methods

#### 3.3.1 Psychometric method

##### 3.3.1.1 Sports environment perception scale

Gong (2021) developed the Physical Education Environment Perception Scale based on College Students’ School Environment Perception Scale (Zhao, 2013) to assess college students’ perceptions of their sport environment. The scale has 19 entries and contains four dimensions: curriculum construction (e.g., question 2: the amount and intensity of practice required by the physical education program is reasonable for you), teacher guidance (e.g., question 6: the teacher adopts inspirational and interactive teaching methods for you in the physical education classroom), institutional environment (e.g., question 13: the physical education guidance and training provided to you by the school you attend is good), and physical environment (e.g., question 15: the physical education atmosphere of the school you attend is) (e.g., Question 15: The physical environment of the school you attend is good). The scale was scored on a 5-point Likert scale from “1” (completely disagree) to “5” (completely agree). The total score of the scale is equal to the sum of the scores of all entries, and the higher the score, the higher the level of college students’ perception of sports environment. In previous studies (Gong et al., 2021), the Sport Environment Perception Scale was used to measure the perception of sport environment among Chinese college students with good reliability and validity. In this study, the Cronbach’s alpha coefficient of the scale was 0.957, and the validated factor analysis fit index was good, indicating that the questionnaire had good reliability and validity in this study.

##### 3.3.1.2 Sports gain scale

The Sport Gain Scale, developed by Gong based on Zhao (2013), was used to test the level of sport gaining of college students (Gong, 2021). The scale has 15 entries and contains 3 dimensions: cognitive gain (e.g., Question 1: Sport participation on your sport professional knowledge and skill gain), practical gain (e.g., Question 9: Sport participation on your ability to use sport practice), and social adaptation gain (e.g., Question 15: Sport participation on promoting your teamwork awareness and ability). The scale is scored on a 5-point Likert scale from “1” (very little improvement) to “5” (a lot of improvement). The total score of the scale is equal to the sum of the scores of all the entries, and the higher the score indicates the greater the gains of college students’ sports. In previous studies (Gong et al., 2021), the Sport Gain Scale was used to measure the sport gain of Chinese college students with good reliability and validity. In this study, the Cronbach’s alpha coefficient of the scale was 0.976, and the validated factor analysis fit index was good, indicating that the questionnaire had good reliability and validity in this study.

##### 3.3.1.3 Physical activity behavior scale

The Physical Activity Rating Scale (PARS-3) developed by Liang (1994) was used to assess the physical activity behavior of college students. The scale contains three modules: activity intensity (1. How intense do you perform physical activity?), activity time, and frequency of exercise, and each module of the scale is scored on a Likert 5-point scale (scale 1–5). The total score is based on the following formula: intensity of exercise*time of exercise*frequency of exercise, with a maximum score of 100 points for physical activity and a minimum of 0. The scores are assessed as follows: small activity≤19 points; smaller activity≤20–39 points; medium activity≤40–59 points; larger activity≤60–79 points; large activity≤80–100 points. In previous studies (Hao et al., 2022), the Physical Activity Behavior Scale was used to measure the physical activity behavior of Chinese college students with good reliability and validity. In this study, the Cronbach’s alpha coefficient of the scale was 0.705, and the validated factor analysis fit index was good, indicating that the questionnaire had good reliability and validity in this study.

##### 3.3.1.4 Sport learning self-efficacy scale

The Sport learning self-efficacy Scale for College Students developed by Luo (2012) was used to measure college students’ Sport learning self-efficacy. The scale consists of Sense of Effort (e.g., Whenever I am in physical education class, I keep myself in a good mood and actively engage in physical education learning), Sense of Control (e.g., I always memorize the key points and difficulties of technical movements in physical education learning, so as to improve my physical education learning ability), and Sense of Competence (e.g., Through physical education learning, I believe that I can improve my physical fitness very well), Sense of Environment (e.g., In the physical education classroom, the more harmonious the relationship between teachers and students is, the better my physical education learning will be) consists of 4 dimensions and contains 20 questions. The scale was scored on a five-point scale (1 for completely disagree, and 5 for completely agree). The total score of the scale is equal to the sum of the scores of all the entries, and the higher the score indicates the higher level of self-efficacy in physical education learning of the subjects. In previous studies (Peng et al., 2012; Deng et al., 2023), the Sport learning self-efficacy Scale was used to measure Chinese college students’ Sport learning self-efficacy, with good reliability and validity. In this study, the Cronbach’s alpha coefficient of the scale was 0.947, and the validated factor analysis fit index was good, indicating that the questionnaire had good reliability and validity in this study.

#### 3.3.2 Mathematical and statistical method

The data of this study were statistically analyzed using SPSS 21.0 software with Process plug-in for SPSS macro program prepared by Hayes. First, descriptive statistics and difference test (*p* < 0.05) were conducted on the test data of demographic information, perception of physical education environment, physical education gain, physical activity behavior and Sport learning self-efficacy using SPSS21.0 software. Second, Harman’s one-way test was used in SPSS26.0 software to test for common method bias. Third, Pearson’s bivariate relationship between college students’ perception of physical education environment, physical education gains, physical activity behavior and Sport learning self-efficacy was tested using SPSS26.0. Fourth, PROCESS plug-in model 6 and Bootstrap (5,000 times) sampling technique were used to test the independent mediating role of physical activity behavior and physical learning self-efficacy, and the chained mediating role between physical environment perception and physical gain. In this study, p < 0.05 was set as statistical result and significant.

## 4 Results

### 4.1 Descriptive statistics of sports environment perception, sports gain, sports exercise behavior, and sports learning self-efficacy

The statistical results in Table 1 show that there is statistical significance (p < 0.05) in the analysis of age and gender differences in sports environment perception, sports gain, sports exercise behavior and sports learning self-efficacy. Boys were higher than girls in the mean values of the test scores of the four variables of sport environment perception, sport gain, sport exercise behavior and sport learning self-efficacy. In addition, the four variables showed a certain regularity in different statistical calibers, which helped this study to further understand the degree of influence and interrelationships of physical education environment perception, physical exercise behavior, and Sport learning self-efficacy on physical education gains.

**Table 1:** Descriptive statistics of the test results of perceived physical education environment, physical education gains, physical exercise behavior and sport learning self-efficacy (x ± SD).

Sex	*n*/numbers	Sport environment perception	Sports gain	Physical activity behavior	Sport learning self-efficacy
Male	903	73.65 ± 10.60	58.04 ± 9.38	55.02 ± 22.45	75.29 ± 10.38
Female	1,304	70.84 ± 11.85	56.37 ± 9.64	50.64 ± 23.29	72.30 ± 11.15
Total	2,207	72.24 ± 11.23	57.20 ± 9.51	52.83 ± 22.87	73.79 ± 10.76
Sex difference (T/p)		5.71/0.00	4.05/0.00	4.41/0.00	6.36/0.00
Age difference (F/p)		17.05/0.00	6.04/0.00	4.82/0.00	5.51/0.00

*P < 0.05 represents significant differences in variables between gender and age.*

### 4.2 Common method bias test

To address potential common methodological bias inherent in questionnaire-based data collection, a proactive measure was taken by employing Harman’s one-factor test (Garrido-Moreno et al., 2021). Through unrotated principal component factor analysis, as recommended by Cowin et al., seven factors with eigenvalues exceeding 1 were extracted. Despite the emergence of seven factors, the primary factor explained only 32.56% of the total variance, falling below the critical 40% threshold (Cowin et al., 2013). This finding implies the absence of substantial common method bias, affirming the dataset’s suitability for subsequent chained mediation effect tests. Consequently, the data appears devoid of significant methodological distortions, supporting its robustness for further analytical investigations.

### 4.3 Correlation analysis of perception of sports environment, gains from sports, physical activity behavior, and self-efficacy in sports learning

As can be seen from Table 2, there is a significant positive correlation between the Sport environment perception, sports gains, physical activity behavior, and self-efficacy in sports learning, and the relationship between the variables supports the subsequent hypothesis test, which provides a better foundation for the mediation effect test in this study.

**Table 2:** Correlation analysis statistics of perceived physical education environment, physical education gains, physical activity behavior and Sport learning self-efficacy.

Variable	Mean scale ± standard deviation	Sport environment perception	Sports gains	Physical activity behavior	Sport learning self-efficacy
Sport environment perception	71.99 ± 11.44	1			
Sports gain	57.05 ± 9.57	0.853**	1		
Physical activity behavior	52.43 ± 23.05	0.693**	0.745**	1	
Sport learning self-efficacy	73.52 ± 10.94	0.744**	0.787**	0.876**	1

*N* = 2,207. **p < 0.001. Same below.

### 4.4 Test of the mediating effect of physical activity behavior and sport learning self-efficacy

**Figure 2:**
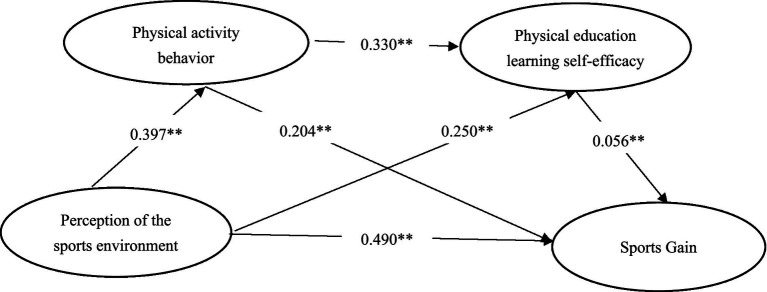
Chain mediation model diagram of physical activity behavior and physical learning self-efficacy between perceived sport environment and sport gains.

Taking the perception of sports environment as the independent variable, physical activity behavior and self-efficacy in sports learning as the mediator variables, and sports gain as the dependent variable, the SPSS macro program plug-in PROCESS provided by Hayes (Hayes, 2017) was used to select model 6 based on Templates, and the bias-corrected nonparametric percentile Bootstrap test was selected (repeated sampling 5,000 times), calculate 95% confidence intervals, and conduct chain mediation model effect analysis (Wen et al., 2022). Therefore, according to Table 3 and the path coefficient result plot (Figure 2), it can be seen that the total effect value of sports environment perception on college students’ sports gain is 0.713 (*t* = 76.60, *p* < 0.001); the path coefficient of sports environment perception on college students’ sports gain is 0.490 (*t* = 39.158, *p* < 0.001); and the path coefficient of sports environment perception on physical activity behavior is 0.397 (*t* = 45.170, *p* < 0.001); the path coefficient of physical activity behavior on sport gains was 0.204 (*t* = 10.399, *p* < 0.001); the path coefficient of physical environment perception on self-efficacy of physical education learning was 0.250 (*t* = 19.984, *p* < 0.001); the path coefficient of self-efficacy of physical education learning on sport gains was 0.056 (*t* = 6.521, *p* < 0.001); the path coefficient of physical activity behavior on Sport learning self-efficacy was 0.330 (*t* = 53.092, *p* < 0.001), and all path coefficients reached the significance level (*p* < 0.001). This result verified the research hypothesis H1.

**Table 3:** Regression analysis of the chain mediation model between perceived sport environment and sport gains.

Variant	Physical activity behavior		Sport learning self-efficacy		Sports Gain		Aggregate effect	
	*β*	*t*	*β*	*t*	*β*	*t*	*β*	*t*
Gender	0.242	17.458**	0.225	15.458**	0.392	54.708**	0.671	71.223**
Age	0.295	23.321**	0.281	20.378**	0.211	13.329**	0.602	59.251*
Sports environment perception	0.397	55.572**	0.250	19.984**	0.490	56.158**	0.713	76.60**
Physical activity behavior			0.330	53.092**	0.204	10.399**		
Sport learning self-efficacy					0.056	6.521**		
*R*2	0.481		0.804		0.783		0.727	
*F*	2040.366		4514.213		2655.631		5867.557	

**P < 0.001.

After the standardized effect value and significance test of the pathway of the perception of sports environment affecting college students’ sports gains, it can be found (Table 4) that the Bootstrap 95% confidence interval of the total indirect effect of the perception of sports environment and college students’ sports gains does not include 0, indicating that there is a significant mediating effect of “physical activity behavior” and “self-efficacy in sports learning” between the perception of sports environment and sports gains (the total indirect effect value is 0.223, and the effect share is 31.28%). The Bootstrap 95% confidence interval does not include 0, indicating that there is a significant mediating effect of “physical activity behavior” and “self-efficacy in physical education” between the perception of physical education environment and physical education gain (the total indirect effect value is 0.223, and the effect ratio is 31.28%). The mediating effect consists of three indirect effects: (1) the indirect effect generated by the path of “sports environment perception” → “physical activity behavior” → “sports gain,” which is the indirect effect of the path of “sports environment perception” → “physical activity behavior” → “sports gain,” which is the indirect effect of the path of “sports environment perception” → “physical activity behavior” → “sports gain.” Bootstrap95% confidence interval does not include 0, indicating that physical activity behavior plays a significant mediating role between the Sport environment perception and sports gain (standardized value = 0.078, accounting for 10.94% of the total effect), which verifies the hypothesis H2; (2) the indirect effect of the pathway “sports environment perception” → “sports learning self-efficacy” → “sports learning self-efficacy” is the same as the indirect effect of the pathway “sports learning self-efficacy” → “sports learning self-efficacy.” (2) the indirect effect generated by the path of “sports learning self-efficacy” → “sports gain,” whose Bootstrap 95% confidence interval does not contain 0, indicating that sports learning self-efficacy plays a significant mediating role between sports environment perception and sports gain (standardized value = 0.078), and this result verifies hypothesis H2 (standardized value = 0.094, accounting for 13.18% of the total effect), this result verifies the hypothesis H3; (3) from “sports environment perception” → “physical activity behavior” → “self-efficacy in sports learning” → “self-efficacy in sports learning” → “self-efficacy in sports learning” → “self-efficacy in sports learning” → “self-efficacy in sports learning” → “self-efficacy in sports learning.” (3) The indirect effect generated by the path of “sports environment perception” → “sports exercise behavior” → “self-efficacy in sports learning” → “sports gain,” whose Bootstrap 95% confidence interval does not contain 0, indicates that sports exercise behavior and self-efficacy in sports learning play a significant role in mediating the chain between sports environment perceptions and sports gains (Normalized value = 0.051, accounting for 7.16% of the total effect. 7.16% of the total effect), and this result tested hypothesis H4.

**Table 4:** Chain mediation effect test of physical activity behavior and physical learning self-efficacy in perceived sport environment and sport gains.

Type of effect	Efficiency value	Boot SE	Bootstrap 95% CI		Efficacy as a percentage of
			lower limit	limit	
Total effect	0.713	0.009	0.695	0.731	100%
Direct effect	0.49	0.013	0.465	0.514	68.72%
Perceived physical activity environment—Physical activity behavior—Physical activity gains	0.078	0.016	0.046	0.111	10.94%
Perceived physical education environment—Sport learning self-efficacy—Physical education gains	0.094	0.014	0.68	0.122	13.18%
Perceived physical education environment—Physical activity behavior—Sport learning self-efficacy—Physical education gains	0.051	0.009	0.034	0.069	7.16%
Total indirect effect	0.223	0.016	0.192	0.254	31.28%

## 5 Discussion

### 5.1 Sports environment perception and sports gaining

The results of this study show that sports environment perception has a significant positive effect on college students’ sport gains, which supported Hypothesis 1. The result is consistent with the results of previous studies (e.g., Gong, 2021; Gong et al., 2021). Several reasons underpin these findings. First, college students’ perceptions of the sport environment play a pivotal role in influencing their overall participation and gains (Hauser et al., 2022). Positive perceptions, including well-maintained facilities, supportive coaches, and an inclusive school climate, serve as motivational catalysts for students, fostering enjoyment and engagement in physical activity. A welcoming physical education environment not only promotes physical fitness but also fosters a stronger sense of identity and builds belonging and involvement in the student-athlete role (De Sisto et al., 2022; Lupo et al., 2017a). Second, a positive and friendly physical activity environment can also increase students’ sport experiences and improve social interaction skills (Wintle, 2022). High-quality facility environments enhance students’ willingness to participate and have a positive impact on their overall sports experience (Kok et al., 2020; Guo et al., 2022). To promote active student participation, it is essential to create an educational environment that encourages involvement and enhances learning. Students can only fully develop their potential by wisely utilizing various university resources and integrating into the university’s environment. Third, curriculum development is vital in school physical education, which affects students’ interest in physical education and the effectiveness of teaching. The physical environment, including infrastructure and funding, shapes the development of school physical education in terms of scale, structure, and content. The quality of stadiums and the level of teachers directly affect the effectiveness of physical education (Dewi et al., 2021). In the university sport environment, the institutional environment plays a leadership role by shaping an enabling ecosystem through policies that are aligned with educational goals. This mechanism, the institutional environment, ensures consistency with student development, rational allocation of resources, and harmonious student-faculty interactions. In essence, the institutional environment promotes the alignment of school sport with broader educational goals and the overall development of students (Gong et al., 2021). It can be seen that increasing the perception of college students’ physical education environment and improving the curriculum construction, teacher guidance, institutional environment, and physical environment can be a practical way to enhance the gains of college students’ physical education. By removing barriers, promoting diversity, and continually improving the sports environment, schools can maximize the positive impact of sports on students’ physical, psychological, and social development, enrich their college life, cultivate their lasting appreciation of an active and healthy lifestyle, and promote their sports gains.

Furthermore, according to the data of the research test of this study, the perception of sports environment and sports gain are statistically significant when analyzing the differences in age and gender. Boys were higher than girls in the mean of the test scores of sports environment perception and sports gain. This is consistent with the results of previous studies (Alcaraz-Muñoz et al., 2023; O’Connor et al., 2023). The reason for this may be that female college students, along with their peers, schools and other factors have not formed a good sports environment atmosphere to motivate their sports participation, with insufficient starting motivation for sports participation, lack of guarantee for sustained conditions, and a lower sense of experience of the results (Gong et al., 2021). Therefore, it is believed that a number of sociocultural factors play a key role in shaping gender differences in sport participation, including social expectations, stereotypes, and historical trends, which contribute to different experiences in the sport environment. Research has also highlighted psychosocial influences, emphasizing the importance of self-confidence, peer influence, and social norms in shaping perceptions and outcomes between genders (Kutuk, 2023). These findings underscore that gender differences in perceptions of the sports environment and sports gains are well established.

In addition, at the level of age differences, developmental factors and changing interests become key factors of differences. Freshmen, for instance, often feel a sense of curiosity about their new surroundings as they transition from the pressures of high school to the more relaxed university environment. They enthusiastically participate in various school and faculty activities, in which sports are the focus of collective integration. Regular sports competitions at all levels are held in which students actively participate and show genuine enthusiasm that deeply inspires them (Evmenenko and Teixeira, 2022). As for the students in higher grades, after adapting to university life, their basic adaptation to the university education, culture, humanities and other environments, freshness and curiosity decrease (Rao et al., 2024). Consequently, university physical education classes and sports activities may fail to sustain their enthusiasm for ongoing participation, as mere novelty is insufficient to maintain long-term engagement (Gong et al., 2021). This indicates that age differences in perceptions of the sports environment and associated gains are also significant. In summary, according to the results of this study, it is feasible and especially important and urgent to target intervention on college students’ sport gains through sport environment perception. Improving the quality of the university sports environment may be one of the ways to promote college students’ sport experience and sport gains.

### 5.2 The mediating role of physical activity behavior

The results of this study show that physical exercise behavior has a mediating role between the perception of sports environment and sports gains, which supported Hypothesis 2. This is consistent with the results of previous studies. Higher levels of physical activity are linked to increased physical fitness, skill development, and overall well-being, which in turn contribute to more favorable sports outcomes for individuals (Panda, 2024). As physical activity behaviors increase, there is a corresponding positive effect on sports gains, reinforcing the notion that an active lifestyle enhances the overall sports experience (Herbert, 2022). As physical activity behaviors increase, there is a corresponding positive effect on sports gains, reinforcing the notion that an active lifestyle enhances the overall sports experience. In addition, positive perceptions of the sports environment are associated with higher participation rates in sports. A conducive sports environment—including convenient facilities and supportive social networks—tends to motivate individuals to engage in sports and adopt an active lifestyle. Positive perceptions of sports environments may lead to increased levels of physical activity among college students, thereby enriching their sports experiences and outcomes (Choi et al., 2021). The health belief model suggests that health-related behaviors are influenced by an individual’s perceptions of the severity of a health threat, susceptibility to a health threat, and the benefits and barriers to taking preventive measures. In terms of participation in sport, individuals who perceive a positive sport environment are more likely to view physical activity behaviors as a valuable and feasible means of improving sport gains, and thus have more positive attitudes toward participation in sport (Bácsné-Bába et al., 2021). The observed mediating effect is consistent with the role of perceived benefits (perceived sport environment) in influencing the likelihood of engaging in health-promoting behaviors (physical activity), which in turn contribute to positive sport outcomes (sport gains), as emphasized by health management theory. This theoretical perspective emphasizes the interconnections between individual perceptions, behaviors, and outcomes in the context of college students’ participation in sport (Boonekamp et al., 2021). These findings highlight the need for stakeholders such as teachers and schools to not only create positive sport environments, but also to understand the critical role that individual behaviors (e.g., regular physical activity) play in translating these perceptions into actual sport outcomes. It can be seen that the latest research literature, both nationally and internationally, supports the findings of this study that physical activity behaviors play a mediating role between perceptions of the sport environment and sport gains. These findings emphasize the importance of improving individuals’ physical activity behaviors by improving their perceptions of the sport environment, thereby improving sport gains.

### 5.3 The mediating role of sport learning self-efficacy

This study also found that sport learning self-efficacy has a mediating role between sport environment perception and sport gains, which supported Hypothesis 3. This aligns with findings from previous research, which indicates a significant positive correlation between sport learning self-efficacy and sports gains (Peers et al., 2020). Bandura’s social cognitive theory suggests that a person’s beliefs about his or her ability to accomplish a particular task (self-efficacy) strongly influences his or her actual performance and subsequent outcomes. In sport, students with higher Sport learning self-efficacy are more likely to confidently tackle learning challenges, skill development, and training, thereby increasing their overall gains in sport (Hovey et al., 2020). That is, an individual’s confidence in his or her ability to learn and improve in sport positively contributes to the overall gains achieved from participation in sport. Furthermore, research has concluded that a positive and supportive learning environment promotes self-efficacy. A conducive sports environment—characterized by accessible facilities, encouraging coaches, and positive peer interactions—can enhance students’ beliefs in their ability to learn and excel in sports activities. This positive perception of the learning environment serves as a foundation for developing self-efficacy in sport (Gong et al., 2021). Bandura’s theory also suggests that a supportive and positive learning environment contributes to the development of self-efficacy beliefs. Elements such as positive feedback, role modelling, and available resources enhance students’ confidence in their abilities to learn and perform in physical education (Leo et al., 2022). This positive perception of the learning environment promotes the development of self-efficacy in physical education learning. As can be seen, Bandura’s social cognitive theory reinforces this notion by emphasizing the importance of environmental factors in shaping self-efficacy beliefs, which in turn influence behavior and outcomes. In this context, a positive learning environment not only contributes directly to increased self-efficacy in physical education learning, but also amplifies the positive impact on physical education gains, thus creating a cascading effect. Thus, the findings emphasize the importance of creating a positive physical education environment that not only promotes skill development, but also fosters self-efficacy and confidence in students’ ability to learn and excel in the field of physical education.

### 5.4 Chain mediation between physical activity behavior and sport learning self-efficacy

The results of this study also found that physical activity behavior and sport learning self-efficacy constitute a chain mediation between sport environment perception and sport gains, which supported Hypothesis 4. This is consistent with the results of previous studies. First, it was concluded that there is a positive correlation between physical learning self-efficacy and physical activity behavior because individuals with higher self-efficacy beliefs are more likely to engage in activities that they find challenging because they are confident in their ability to overcome difficulties (Ten Brummelhuis et al., 2022). In the case of physical education, students with higher self-efficacy for physical education learning are more likely to participate in a variety of physical activities because they know that they have sufficient skills and abilities to accomplish these physical activities (Clevinger et al., 2020). Second, scholars have noted that higher self-efficacy not only affects physical activity participation, but also positively impacts learning outcomes. Students with higher self-efficacy are more likely to meet learning challenges with persistence and enthusiasm, resulting in improved skill acquisition and overall physical education learning outcomes (physical education gains). Bandura’s theory suggests that individuals with strong beliefs in their ability to learn and perform in sport are more likely to achieve positive outcomes, thus establishing a link between self-efficacy, behavior, and ultimate gains (Yılmaz et al., 2020). Finally, research has demonstrated that a supportive and positive learning environment boosts students’ confidence in their abilities, which subsequently influences their participation in physical activity and their sense of efficacy throughout the learning process. Moreover, self-efficacy beliefs during this process affect behaviors and outcomes, including sports gains. This aligns with Bandura’s concept of reciprocal determinism, which emphasizes the dynamic interaction among environmental factors, individual behaviors, and personal attributes (Rumawatine, 2023). It can be seen that sport environment perception improves sport learning self-efficacy by increasing individual physical activity behavior, which in turn improves sport gains. The findings of this study highlight the critical roles of physical activity behavior and sport learning self-efficacy in helping college students achieve greater sports outcomes. Together, these factors form a chain mediating the relationship between sport environment perception and sports gains. Furthermore, the perception of the physical education environment is deeply reflected in elements such as curriculum design, instructor guidance, support services, and cultural incentives. These elements significantly influence students’ participation in physical education and serve as key facilitators for enhancing the quality of physical education teaching. Therefore, it is suggested that in the future, in order to enhance the level of sport gains of university students, their physical activity behavior can be enhanced by encouraging a positive perception of the sport environment and fostering good self-efficacy for sport learning, which in turn will promote the sport gains of university students.

## 6 Research shortcomings and outlook

First, this study explored the influencing factors of college students’ sport gains and proposed the role of sport environment perception and sport learning self-efficacy in increasing physical activity behavior and promoting sport gains. It has important theoretical value for understanding the causes of college students’ sport gains, and also provides inspiration for the prevention and intervention of college students’ sport gains. Our findings contribute to the existing body of knowledge by providing empirical support for the role of perceived sport environment in sport gain management. This highlights the importance of considering sport environment perception and sport gaining in understanding the complex interplay between physical activity behavior and sport learning self-efficacy. It also highlights the potential benefits of incorporating assessments of perceived physical activity environments into future sport gaining intervention studies. The findings of this study have practical help implications for physical education teachers and educational institutions to recognize the impact of perceived sport environment on sport gains, and targeted interventions can be developed to implement programs that promote positive sport experiences, such as providing a variety of enjoyable sport activities and creating supportive environments, which may help to promote sport participation and its gains among college students.

Second, at the school level, gender differences should be taken into account and schools should focus on creating a positive physical education environment. Tailoring the curriculum to different interests, promoting inclusivity, and raising awareness of the long-term benefits of physical activity may enhance the student experience; from the perspective of the physical education teacher, physical education teachers should provide individualized support to address gender-related differences and create a positive environment. Introducing diverse activities and maintaining open communication with parents can ensure a more engaging and supportive learning environment; From the parents’ perspective, parents should encourage their children to be physically active at home. Parents should encourage their children to be physically active at home, advocate for inclusivity in the school program, and foster a positive mindset. Supporting a diverse and inclusive physical education program ensures a welcoming environment for all students; From the individual college student’s perspective, students should reflect on their perceptions, seek support to increase their self-efficacy, and diversify their physical activity for a more positive experience. Promoting inclusivity in a school’s athletic culture can help create a mutually supportive environment for everyone.

However, while this study reveals the relationship between perceived sport environment and sport gains, its limitations must be recognized. First, the cross-sectional design precludes causal inferences, and thus experimental interventions or longitudinal studies need to be considered to explore mechanisms and temporal dynamics more fully. Second, reliance on a single investigative instrument may be biased by common differences in methodology. Therefore, future studies should utilize multiple assessment tools. Additionally, the focus of our study on college students at a specific university raises concerns about generalizability, and thus the use of objective assessment methods and a broader sample are needed to improve reliability. To address subjective bias in self-report data, it is recommended that intrinsic mechanisms and psychosocial factors, such as social and peer support, be explored in future studies. In conclusion, the use of different assessment tools, different research designs, increased sample sizes, the use of objective measures, and the exploration of other influencing factors are critical to deepening our understanding and refining the methods used to manage college students’ sport gains.

## 7 Conclusion

1. Perception of physical education environment, gains from physical education, physical activity behavior and self-efficacy for physical education learning were statistically significant when analyzed for age and gender differences. Male students had higher mean scores than female students in the four variables of perceived physical activity environment, physical activity gains, physical activity behavior and physical activity learning self-efficacy.
2. Perception of sports environment plays an important role in predicting college students’ physical activity behavior, self-efficacy in physical education and gains in physical education. A good Sport environment perception can help to improve individuals’ physical activity behavior, make them more active in sports and achieve excellent results. Good sport learning self-efficacy also enhances individuals’ physical activity behavior. The increase in physical activity behavior and self-efficacy for physical education and the increase in perception of the physical education environment can promote college students’ sport gains. Perceptions of the sport environment are predictive of physical activity behavior, sport learning self-efficacy, and sport gains.

## Data Availability

The raw data supporting the conclusions of this article will be made available by the authors, without undue reservation.
