# Supplementary material for: The effects of perceived sport environment on sport gains of Chinese university students: chain mediation between physical activity behavior and sport learning self-efficacy
Source: Front Psychol. 2024 Dec 10;15:1466457. doi: 10.3389/fpsyg.2024.1466457 (PMC11668181; doi:10.3389/fpsyg.2024.1466457)
Supplement: Supplementary file 1 [file Table_1.DOCX]

**Perceived Athletic Environment Scale**

The following questions reflect the environment and services provided by the school for you to participate in sports, please answer according to your actual experience.

Please answer according to your actual experience. A score of 5 indicates that the quality of the service provided by the school is "very good", while a score of 1 indicates that it is "very poor".

| serial number | Issue entry | totally agree | relatively agree | general | disagree | Strongly disagree. |
| --- | --- | --- | --- | --- | --- | --- |
| 1 | The physical education program stimulates your interest in learning | 5 | 4 | 3 | 2 | 1 |
| 2 | Physical education programs require a reasonable amount and intensity of practice for you | 5 | 4 | 3 | 2 | 1 |
| 3 | The PE program requires your active participation inside and outside the classroom | 5 | 4 | 3 | 2 | 1 |
| 4 | The physical education program's practical aspects are reasonable for you | 5 | 4 | 3 | 2 | 1 |
| 5 | Physical education emphasizes the development of your independent thinking skills. | 5 | 4 | 3 | 2 | 1 |
| 6 | Teachers use inspirational and interactive teaching methods in the PE classroom | 5 | 4 | 3 | 2 | 1 |
| 7 | Teachers emphasize the development of your ability to apply your knowledge and skills in sport | 5 | 4 | 3 | 2 | 1 |
| 8 | Teachers provide you with opportunities to participate in your own organized sports activities | 5 | 4 | 3 | 2 | 1 |
| 9 | Teachers respond positively to your new ideas or questions. | 5 | 4 | 3 | 2 | 1 |
| 10 | Teachers guide you to participate in athletic competitions and self-directed physical activity. | 5 | 4 | 3 | 2 | 1 |
| 11 | How well your school provides you with psychological counseling services and guidance | 5 | 4 | 3 | 2 | 1 |
| 12 | Your school provides you with effective channels to express your opinions and gives you timely feedback. | 5 | 4 | 3 | 2 | 1 |
| 13 | Your school provides you with good sports guidance and training. | 5 | 4 | 3 | 2 | 1 |
| 14 | The academic atmosphere at your school is good | 5 | 4 | 3 | 2 | 1 |
| 15 | The atmosphere of your school is good | 5 | 4 | 3 | 2 | 1 |
| 16 | The living conditions at your school are good. | 5 | 4 | 3 | 2 | 1 |
| 17 | Your school's campus network is well developed and utilized. | 5 | 4 | 3 | 2 | 1 |
| 18 | Your school provides you with excellent conditions for participation in sports. | 5 | 4 | 3 | 2 | 1 |
| 19 | Your school's athletic facilities are well resourced and easy to use | 5 | 4 | 3 | 2 | 1 |
