# Supplementary material for: The effects of perceived sport environment on sport gains of Chinese university students: chain mediation between physical activity behavior and sport learning self-efficacy
Source: Front Psychol. 2024 Dec 10;15:1466457. doi: 10.3389/fpsyg.2024.1466457 (PMC11668181; doi:10.3389/fpsyg.2024.1466457)
Supplement: Supplementary file 2 [file Table_2.DOCX]

Physical Activity Rating Scale (PARS-3)

The following questions measure how physically active you have been in the last month. For the first three questions, choose the one that fits best and write the answer number in parentheses after the question, and for question 4, underline the program in which you engaged in exercise or write your exercise program.

1、What is the intensity of your physical exercise?

① Light exercise (e.g., walking, doing radio gymnastics, playing goalball, etc.)

② small intensity of less intense sports (such as recreational volleyball, table tennis, jogging, tai chi, etc.)

③ moderate intensity of the more intense and long-lasting exercise (such as cycling, running, playing table tennis, etc.)

④High-intensity but not long-lasting sports with shortness of breath and sweating (e.g. playing badminton, basketball, tennis, soccer, etc.)

(5) High-intensity, long-lasting sports that involve a lot of shortness of breath and sweating (e.g., running, aerobics, swimming, etc.).

2、How many minutes at a time do you perform the above intensity sports activities?

① Less than 10 minutes

②11 to 20 minutes

③21 to 30 minutes

③21 to 30 minutes ④31 to 59 minutes

⑤More than 60 minutes

3. How many times do you do the above sports activities?

① Less than once a month

② 3 to 5 times a week

③2 to 3 times a month

③2 to 3 times a month ④Approximately 1 time a day

4、 What kind of sports do you like?

①walking, running

②Traveling, picnicking

③Sports dance

④Ball games

⑤ Rope skipping

⑥ Taijiquan, health techniques

⑦ Fitness equipment activities

⑧ Swimming

Translated with www.DeepL.com/Translator (free version)
