# Supplementary material for: The effects of perceived sport environment on sport gains of Chinese university students: chain mediation between physical activity behavior and sport learning self-efficacy
Source: Front Psychol. 2024 Dec 10;15:1466457. doi: 10.3389/fpsyg.2024.1466457 (PMC11668181; doi:10.3389/fpsyg.2024.1466457)
Supplement: Supplementary file 3 [file Table_3.DOCX]

**Self-efficacy in Physical Education for College Students.**

Scoring method: The sum of the scores of the above questions is the self-efficacy score of college students in physical education, and the higher the score, the higher the self-efficacy level of the subjects. The Physical Education Self-Efficacy Scale is a self-stated scale. The scale consists of a total of 20 test items, and the scale is based on a five-point scale. Its reverse question responses are not at all (1), not at all (2), fair (3), fairly (4), and completely (5)

| No. | Question Entry | Completely Compliant | Comparatively Compliant | General | Comparatively non-compliant | Not at all |
| --- | --- | --- | --- | --- | --- | --- |
| 1 | Whenever I am in physical education class, I keep myself in a good mood and actively participate in physical education. | 5 | 4 | 3 | 2 | 1 |
| 2 | When I am criticized by the teacher, I can control my emotions and continue practicing. | 5 | 4 | 3 | 2 | 1 |
| 3 | After practicing for a long time, I can still control myself not to be distracted and focus on the classroom tasks. | 5 | 4 | 3 | 2 | 1 |
| 4 | When practicing new techniques, I can follow the teacher's instructions to the letter. | 5 | 4 | 3 | 2 | 1 |
| 5 | No matter how difficult the tasks in PE class are, I will try my best to complete them. | 5 | 4 | 3 | 2 | 1 |
| 6 | Even if I don't do well in a sports test, I believe that if I work harder, I will do better next time. | 5 | 4 | 3 | 2 | 1 |
| 7 | I always write down the key points and difficulties of the technical movements in physical education, so as to improve my ability to learn physical education. | 5 | 4 | 3 | 2 | 1 |
| 8 | I always try my best to think and practice when I learn new techniques. | 5 | 4 | 3 | 2 | 1 |
| 9 | I always ask my physical education teacher for some knowledge about sports. | 5 | 4 | 3 | 2 | 1 |
| 10 | Whenever I encounter difficulties or setbacks in learning new skills, I will adjust myself and try my best to overcome them. | 5 | 4 | 3 | 2 | 1 |
| 11 | I will take the initiative to ask my teacher or classmates for help when I encounter difficult sports movements. | 5 | 4 | 3 | 2 | 1 |
| 12 | I believe that I can understand the complex sports skills taught by the teacher in physical education. | 5 | 4 | 3 | 2 | 1 |
| 13 | I believe I can improve my physical fitness through physical education. | 5 | 4 | 3 | 2 | 1 |
| 14 | If a skill is difficult to understand, I believe I can figure it out. | 5 | 4 | 3 | 2 | 1 |
| 15 | I find it difficult to learn some of the techniques that require high body coordination. | 5 | 4 | 3 | 2 | 1 |
| 16 | If my classmates who are similar to me in terms of foundation score better than me in physical education, I believe I will surpass them in the future. | 5 | 4 | 3 | 2 | 1 |
| 17 | I think the equipment and facilities for physical education are outdated and not conducive to classroom practice. | 5 | 4 | 3 | 2 | 1 |
| 18 | The better the teacher-student relationship is in the PE classroom, the better my PE learning will be. | 5 | 4 | 3 | 2 | 1 |
| 19 | I want to continue to do well in PE class even when the weather is bad | 5 | 4 | 3 | 2 | 1 |
| 20 | The strict rules of the physical education teacher in the classroom can motivate me to participate in exercise better. | 5 | 4 | 3 | 2 | 1 |
