# Supplementary material for: The effects of perceived sport environment on sport gains of Chinese university students: chain mediation between physical activity behavior and sport learning self-efficacy
Source: Front Psychol. 2024 Dec 10;15:1466457. doi: 10.3389/fpsyg.2024.1466457 (PMC11668181; doi:10.3389/fpsyg.2024.1466457)
Supplement: Supplementary file 4 [file Table_4.DOCX]

**Sport Gain Scale**

The following questions reflect the improvement and development of your sports knowledge and skills.Please answer as appropriate, with 5 indicating "a great deal" and 1 indicating "very little".

| No. | Problem entry | Very much improved | Highly improved | Fairly improved | improved Lowly | Very little improved |
| --- | --- | --- | --- | --- | --- | --- |
| 1 | Sport participation gains on your sport expertise and skills | 5 | 4 | 3 | 2 | 1 |
| 2 | The impact of sport participation on your ability to apply what you have learned about sport to your sporting activities. | 5 | 4 | 3 | 2 | 1 |
| 3 | Participation in sport has improved your ability to collect information about sport through effective channels. | 5 | 4 | 3 | 2 | 1 |
| 4 | The impact of participation on your sense of creativity and your ability to take the initiative to innovate in the practice of sport learning. | 5 | 4 | 3 | 2 | 1 |
| 5 | The impact of sports participation on your ability to learn and practice sports on your own. | 5 | 4 | 3 | 2 | 1 |
| 6 | The impact of sport participation on your ability to appreciate sport | 5 | 4 | 3 | 2 | 1 |
| 7 | The impact of your participation on your international outlook on sports issues | 5 | 4 | 3 | 2 | 1 |
| 8 | The impact of your participation on your ability to express theories in sport | 5 | 4 | 3 | 2 | 1 |
| 9 | Your ability to apply sport in practice | 5 | 4 | 3 | 2 | 1 |
| 10 | The impact of sports participation on your independent opinion and correct value judgment on sports. | 5 | 4 | 3 | 2 | 1 |
| 11 | The impact of your participation on your positive attitude towards life. | 5 | 4 | 3 | 2 | 1 |
| 12 | The impact of sports participation on your ability to recognize yourself correctly and objectively | 5 | 4 | 3 | 2 | 1 |
| 13 | Your sense of social and family responsibility | 5 | 4 | 3 | 2 | 1 |
| 14 | Participation in sport promotes your ability to communicate effectively and get along well with different kinds of people. | 5 | 4 | 3 | 2 | 1 |
| 15 | Sports participation promotes your sense of teamwork and your ability to work together | 5 | 4 | 3 | 2 | 1 |
